# Callous-unemotional traits and externalizing problem behaviors in left-behind preschool children: the role of emotional lability/negativity and positive teacher-child relationship

**DOI:** 10.1186/s13034-023-00633-8

**Published:** 2023-06-29

**Authors:** Ruifeng Tan, Xinying Guo, Suiqing Chen, Guixian He, Xingtao Wu

**Affiliations:** 1 School of Education, Guangzhou University, Guangzhou, China; 2 Luoding Secondary Vocational Technical school, Yunfu, China

**Keywords:** Callous-unemotional traits, Externalizing problem behaviors, Left-behind, Emotional lability/negativity, Teacher-child relationship

## Abstract

**Background:**

Callous-unemotional traits and emotional lability/negativity of young children have been regarded as the markers of externalizing problem behaviors. Based on the sensitivity to threat and affiliative reward model and the general aggression model, emotional lability/negativity may act as a mediator in the relationship between callous-unemotional traits and externalizing problem behaviors. Additionally, a positive teacher-child relationship could act as a buffer given the parental absence in left-behind children. However, these links remain unexplored in left-behind preschool children. Therefore, this study explored the link between callous-unemotional traits of left-behind preschool children and externalizing problem behaviors, as well as the mediating role of emotional lability/negativity and the moderating role of a positive teacher-child relationship.

**Method:**

Data were collected on 525 left-behind children aged 3 to 6 years from rural kindergartens in China. Preschool teachers reported all data through an online survey platform. Moderated mediation analysis was performed to examine whether the mediated relation between callous-unemotional traits and externalizing problem behaviors was moderated by a positive teacher-child relationship.

**Results:**

The results showed callous-unemotional traits significantly predicted externalizing problem behaviors and lability/negativity acted as a mediator, while a positive teacher-child relationship acted as a protective factor in moderating the relationship between callous-unemotional traits and emotional lability/negativity. This study identified a moderated mediation effect among the four variables in left-behind preschool children in China.

**Conclusion:**

The findings provide support for the advancement of theoretical foundations, and provide an avenue for further exploration to support the mental health and overall development of left-behind children during early childhood.

## Introduction

In China, left-behind children (LBC) emerged with the economic and social development of the country. Since the reform and opening up, the uneven development of the region has led to large-scale migration and mobility of labor. LBC emerged when migrant parents left their children in their place of origin to be cared for by grandparents or other guardians. Preschool LBC mainly refer to children whose parents have been working abroad for a long time before the age of 6 and have not yet received compulsory education [1], which is about 20% or more of the total number of LBC in China [2]. The neglect of LBC by migrated parents has had an emotional impact on these children [3]. The insecure attachments caused by less parental supervision and overbearing or overly indulgent care from other guardians may prompt the excessive introversion or egotism of LBC emotionally [4], and more pronounced behavioral problems [5]. Early childhood is a critical stage in a child’s emotional and social development, providing an important foundation for future school adjustment and positive interpersonal relationships [6].

The general aggression model (GAM) proposes that conduct disorders is the outcome of environmental factors and self-developmental factors acting on the proximal psychological status of children [7]. In consideration of the findings in previous empirical studies, negative personality traits (e.g., neuroticism and psychoticism) of LBC were more prominent than in non-left-behind children in middle childhood [8]. Studies have found that impulsive personality traits are associated with emotional incompetence [9], and LBC with absent parents showed emotional inability and anxiety [10]. Similarly, callous-unemotional (CU) traits have been proposed as a predictor of conduct disorder, which is considered externalizing behavior as opposed to internalized negative emotional states such as low emotional responsiveness [11]. Based on the GAM, the role of environmental factors cannot be ignored, especially the protective and buffering effects. For example, while the parents of LBC have migrated, a child’s teacher can serve as an attachment figure and play a protective role buffering the effects of negative personality traits, which may be more prominent in LBC, on emotional and behavioural adaptation [12, 13]. However, it remains unclear as to the mechanisms that may link certain individual characteristics or traits to conduct disorders in LBC in early childhood.

### Callous-unemotional traits and externalizing problem behaviors

Externalizing problem behaviors (EPBs) refer to an individual’s explicit and negative out-of-control behaviors, such as aggression, destructiveness, resistance, hyperactivity, and impulsivity [14]. CU traits have been regarded as contributing to EPBs of preschool children [15, 16]. CU traits refer to the personality tendency of low sensitivity to reward and punishment, as well as low empathy or high indifference to others [17]. Based on ecosystem theory, EPBs in LBC have always been a concern [18, 19], especially the effect of individual characteristics on EPBs [20]. Eysenck’s biological theory emphasized the importance of personality in early childhood [15, 21], and the relationship between certain personality traits and externalizing symptoms has been identified among LBC [22]. For example, Children with high CU traits mainly showed a low level of sensitivity to rewards and punishment, and were more likely to exhibit impulsive or destructive behaviors as well [23]. Additionally, previous studies indicated that CU traits uniquely predicted externalizing symptoms (e.g. conduct problems and oppositional behavior) in preschool children [24–26]. The previous study have examined the close relationships among insecure parent-child attachment, CU traits and conduct problems [27].

Therefore, young preschool LBC should deserve more attention. It can be surmised that the higher the CU traits, the more likely LBC will exhibit EPBs. Therefore, this study proposed the following hypothesis.

#### Hypothesis 1

CU traits in left-behind children during early childhood would significantly positively predict EPBs.

### Emotional lability/negativity as a mediator

As one of the important indicators of socioemotional development, emotional lability/negativity (LN) refers to children’s reacting rapidly to cues that trigger emotions and having difficulty recovering from adverse emotional reactions [28, 29]. Higher levels of LN in children are associated with lower levels of social adaptation [30]. Children’s emotional LN would be impacted by many internal and external factors, including personality traits [31]. Emotional LN co-occurs with CU traits in people with externalizing problems generally [32]. The triarchic model of psychopathy proposes that disinhibition is related to impulsivity and negative affectivity [33]. Studies demonstrated that children’s CU traits correlated with instability in emotional functioning [34, 35]. Individuals with maladaptive personality traits may have difficulty controlling high levels of negative emotions, which could further lead to EPBs [36]. Accordingly, children with high CU traits may have more intense emotional responsiveness that is self-oriented, instead of emotional responses that are other-oriented [11]. The relationships between CU traits and emotional intensity and resilience might be more evident if the child has experienced maltreatment and psychological distress [37]. Furthermore, the literature showed the fluctuating status in emotions of LBC who were left for a long period might be linked to psychoticism [38]. This finding suggests a possible association between CU traits and emotional LN in LBC during early childhood.

GAM suggests that emotional status could play a key role in the nexus of personality traits and externalizing behaviors [7]. The sensitivity to threat and affiliative reward (STAR) model proposes that children in an at-risk context (e.g., maltreatment) would show “reactionary callousness” and experience negative emotionality [39]. Children with early EPBs and high CU traits often have the pattern of negative emotional lability and shifts [40, 41]. Furthermore, emotional LN is regarded as a marker of EPBs in preschoolers [42]. Emotional LN may be the upfront manifestation of EPBs effected by high CU traits, such as the symptoms of LBC [20]. Therefore, this study proposed the following hypothesis.

#### Hypothesis 2

Emotional LN plays a mediating role in the relationship between CU traits and EPBs in left-behind children during early childhood.

### Positive teacher-child relationship as a moderator

Based on ecosystem theory, children’s development is related to multiple contextual factors [18, 43], such as the teacher-child relationship (TCR) [44, 45]. TCR refers to the psychological multisystem formed between young children and teachers in kindergarten, with emotional, cognitive, and behavioral interactions as the main manifestations [46]. As a contextual factor, TCR could positively predict preschool children’s social and emotional adjustment [47]. The goodness-of-fit model proposes that children’s temperaments interact with their external contextual factors to affect children’s development [48]. As such, a positive TCR would partially compensate for the negative effects of CU traits [49]. For instance, a positive association between CU traits and punishment insensitivity to teachers has been found [50], as well as a negative relationship between a positive TCR and CU traits [51, 52]. Meanwhile, an empirical study found the interactive effect of these two variables on emotional and behavioral adjustment in preschool children [53]. Based on the GAM, an interactive effect of contextual factors and personality traits on internal emotional states can be proposed [7]. One study found that the TCR had a moderating impact on the relationship between temperamental characteristics and emotional functioning [54]. Additionally, there was a significant pairwise connection among TCR, CU traits, and emotional LN in children [35].

From the contribution of attachment theory to positive TCR, the teacher was perceived as an unique attachment figure who could provide a safe haven and the function of seeking comfort for young children [55]. TCR quality played a buffering role in the positive association between poor parental monitoring and low emotional control in children [56]. LBC may lack opportunities for parent-child interaction, while teachers, as an important attachment figure, may have a positive effect on the development of children’s prosocial emotions by forming a positive TCR [12, 57]. Though the number of studies limited, existing studies implied that positive TCR might reduce CU trait development increasingly [58–60], especially for children with insecure attachment experiences [61]. Therefore, this study proposed the following hypothesis.

#### Hypothesis 3

A positive TCR plays a moderating role in the link between CU traits and emotional LN in left-behind children during early childhood.

### The current study

In order to explore the underlying mechanisms in the association between CU traits and EPBs in left-behind children during early childhood, this study explored the mediating role of LN and the moderating role of TCR. Specifically, the following three hypotheses were tested: (1) CU traits would significantly positively predict EPBs, (2) emotional LN plays a mediation effect in the relationship between CU traits and EPBs, and (3) a positive TCR plays a moderating role in the relationship between CU traits and emotional LN. The results of the current study could provide empirical support for the GAM, a better understanding of factors associated with the social adaptation of LBC in early childhood, and a comprehensive perspective to promote their emotional and behavioral development.

## Methods

### Participants and procedure

Purposeful or convenience sampling was used to recruit kindergarten teachers of LBC aged 3–6 in rural areas of Guangdong province, China. A total of 638 questionnaires were distributed through Wenjuanxing as the Chinese online survey platform (http://www.wjx.cn, accessed on Jan 15, 2023). As the attachment figure of LBC, kindergarten teachers report all data. After excluding invalid questionnaires (e.g., missing some items, short duration and answer inconsistency obviously), 525 were included in the analysis (response rate of 82.3%). Among them, 265 were boys (50.50%, M_age_=4.20, SD = 0.81) and 260 girls (49.50%, M_age_=4.22, SD = 0.87). The study was reviewed approved by the research ethics committee of Guangzhou University (Protocol Number: GZHU202301).

### Measures

#### Callous-unemotional traits

The Inventory of Callous-Unemotional Traits (ICU) [62] was used to evaluate CU traits. The Chinese short version has been revised and includes two dimensions: uncaring and callousness [63]. The questionnaire has 11 items (e.g., He/she seems cold and inconsiderate) that are responded to using a 4-point scale (scored 1–4). The higher the score, the higher the degree of CU traits. Previous studies have shown the scale has great reliability and validity in Chinese preschool children [64, 65]. In this study, the Cronbach’s α of the ICU was 0.78, KMO = 0.84, and the Bartlett test *p* < 0.001.

#### Emotional lability/negativity

Emotional LN was measured using the Emotional Lability/Negativity Scale [66]. In this study, the revised Chinese version of this scale was used to assess emotional LN, which consists of 7 items that are responded to using a 4-point scale [67]. The items primarily assess emotional flexibility, dysregulation, and unpredictability of negative emotions. The scale has demonstrated good reliability and validity in previous research with Chinese preschool children [68]. In this study, Cronbach’s α was 0.88, KMO = 0.90, and Bartlett test *p* < 0.001.

#### Externalizing problem behaviors

According to existing studies [69–71], EPBs was measured based on the two scales of the Strength and Difficulties Questionnaire (SDQ) (i.e. conduct problems and hyperactivity/inattention). Each scale has five items (conduct problems: e.g., often fights, lies or cheats, and hyperactivity/inattention: e.g., restless, overactive, unable to stay still for long) that are responded to using a 3-point scale (scored 0–2 points). Higher scale scores indicate more externalizing behaviors. Previous studies indicate the SDQ has good reliability and validity [72]. In this study, Cronbach’s α of the subscale was 0.73, KMO = 0.83, and the Bartlett test *p* < 0.001.

#### Teacher-child relationship

The TCR was evaluated using the Chinese version of the Student-Teacher Relationship Scale (STRS) [73, 74], which consists of 28 items that are responded to using a 5-point scale (scored 1–5 points). Due to the low reliability of the Dependency subscale in the Chinese social context, only the subscales assessing teacher-child closeness and teacher-child conflict were used [75]. The Conflict (8 items) and Closeness (7 items) subscales consist of 15 items total. Higher scores indicate a more positive teacher-child relationship. Previous studies have demonstrated that the 15-item STRS has good reliability and validity [76]. In this study, Cronbach’s α = 0.86, KMO = 0.91, and the Bartlett test *p* < 0.001.

### Data processing and analysis

All statistical analysis of data was conducted by SPSS 26.0. and its macro program. First, a Pearson correlation matrix that included young LBC’s CU traits, emotional LN, EPBs, and positive TCR was constructed. Second, two macro-Model were used in the further analysis [77]. According to the hypothesis 1 and 2, PROCESS Model 4 was performed to examine the mediating effect of emotional LN on the link between CU traits and EPBs. The moderating role of TCR in the link of CU traits and emotional LN was tested via PROCESS Model 7. Third, in the parameter test, the Bootstrap method was used to test the significance of the regression coefficient, a total of 5000 samples were constructed, each sample size was 525. The standard deviation and confidence interval of the parameter estimation were obtained. If the 95% confidence interval does not include 0, the result is significant, and vice versa [78].

## Results

### Preliminary analyses

The results of the difference test showed that there were gender differences in CU traits (*t* = 2.34, *p* < 0.05, *Cohen’s d* = 0.20), EPBs (*t* = 2.49, *p* < 0.05, *Cohen’s d* = 0.22), and the TCR (*t* = -2.16, *p* < 0.05, *Cohen’s d* = 0.19). There was also a significant age difference in CU traits: older age was associated with less CU traits (*F* = 3.43, *p* < 0.05, *η*^*2*^ = 0.01). For the precision of the analysis, gender and age were regarded as control variables in subsequent examinations to exclude their effects.

The results of the Pearson correlation analysis are shown in Table 1. CU traits significantly positively correlated with emotional LN (*r* = 0.54, *p* < 0.01) and EPBs (*r* = 0.64, *p* < 0.01), and emotional LN positively correlated with EPBs (*r* = 0.61, *p* < 0.01). In contrast, a positive TCR was negatively associated with CU traits (*r* = -0.69, *p* < 0.01), emotional LN (*r* = -0.46, *p* < 0.01), and EPBs (*r* = -0.56, *p* < 0.01). Thus, higher CU traits in preschool LBC was associated with higher levels of emotional LN and EPBs, whereas a positive TCR was linked with reduced levels of emotional LN and EPBs.

**Table 1:** Descriptive statistics and correlations among the variables

Variables	M	SD	1	2	3	4	5	6
1 Gender	0.51	0.50	1					
2 Age	4.21	0.84	-0.01	1				
3 CU traits	2.07	0.41	0.10^*^	-0.09^*^	1			
4 Emotional LN	1.77	0.56	0.07	-0.03	0.54^**^	1		
5 EPBs	0.58	0.32	0.11^*^	-0.04	0.64^**^	0.61^**^	1	
6 Positive TCR	3.86	0.62	-0.09^*^	-0.01	-0.69^**^	-0.46^**^	-0.56^**^	1

N = 525. Gender was a virtual encoding variable, Boy = 1, Girl = 0. CU traits, callous-unemotional traits; Emotional LN, emotional lability/negativity; Positive TCR, positive teacher-child relationship; EPBs, externalizing problem behaviors

^*^p < 0.05. ^**^p < 0.01

### Testing for a mediation effect

In order to reveal the mediating role of emotional LN in the relationship between CU traits and EPBs, multiple regression was used controlling for the effects of gender and age. The results showed CU traits significantly positively predicted externalizing problem behaviors (*β* = 0.54, *p* < 0.01). Next, the Model 4 macro program was selected to construct a mediation model with emotional LN as a mediator. The direct effect of CU traits on EPBs was significant (Direct effect = 0.44, SE = 0.04, Boot CI = [0.37, 0.52]) and the indirect effect of emotional LN was significant (Indirect effect = 0.20, SE = 0.03, Boot CI = [0.15, 0.25]). The effect size of the mediating effect, i.e. the contribution rate of mediation effect in emotional LN expressed as the ratio of indirect effect to total effect, was about 31%. Therefore, emotional LN played a mediating role in the link between CU traits and EPBs.

### Testing for moderated mediation

To further investigate whether the TCR plays a moderating role between CU traits as the predictor and emotional LN as the mediator, Macro-Model 7 was selected to test the moderated mediation effect. After controlling for the effects of gender and age, the results (as shown in Table 2) showed that CU traits positively predicted EPBs (*β* = 0.44, *p* < 0.01), emotional LN positively predicted EPBs (*β* = 0.36, *p* < 0.01), and CU traits had a significant positive predictive effect on emotional LN (*β* = 0.43, *p* < 0.01). However, positive TCR negatively predicted emotional LN (*β* = -0.17, *p* < 0.01). Moreover, the interaction effect of CU traits and TCR negatively predicted emotional LN (*β* = -0.08, *p* < 0.05). Thus, the TCR moderated the link of CU traits and emotional LN.

**Table 2:** Evaluating the moderated mediation effects: emotional LN as the moderator

	Model 1: Emotional LN				Model 2: EPBs			
	*β*	*SE*	*t*	*95% CI*	*β*	*SE*	*t*	*95% CI*
CU traits	0.43	0.51	8.44^**^	[0.33, 0.53]	0.44	0.04	12.12^**^	[0.37, 0.52]
Positive TCR	-0.17	0.05	-3.36^**^	[-0.27, -0.07]				
CU traits × Positive TCR	-0.08	0.03	-2.48^*^	[-0.15, -0.02]				
Emotional LN					0.36	0.04	9.98^**^	[0.29, 0.44]
Gender	0.02	0.07	0.24	[-0.13, 0.16]	0.08	0.06	1.27	[-0.04, 0.20]
Age	-0.01	0.05	-0.10	[-0.10, 0.09]	0.01	0.04	0.34	[-0.06, 0.09]
*R* ^*2*^	0.31				0.51			
*F*	47.53^**^				135.03^**^			

N = 525. Gender and age were controlled as covariates. CU traits, callous-unemotional traits; Emotional LN, emotional lability/negativity; Positive TCR, positive teacher-child relationship; EPBs, externalizing problem behaviors

^*^p < 0.05. ^**^p < 0.01

**Fig. 1:**
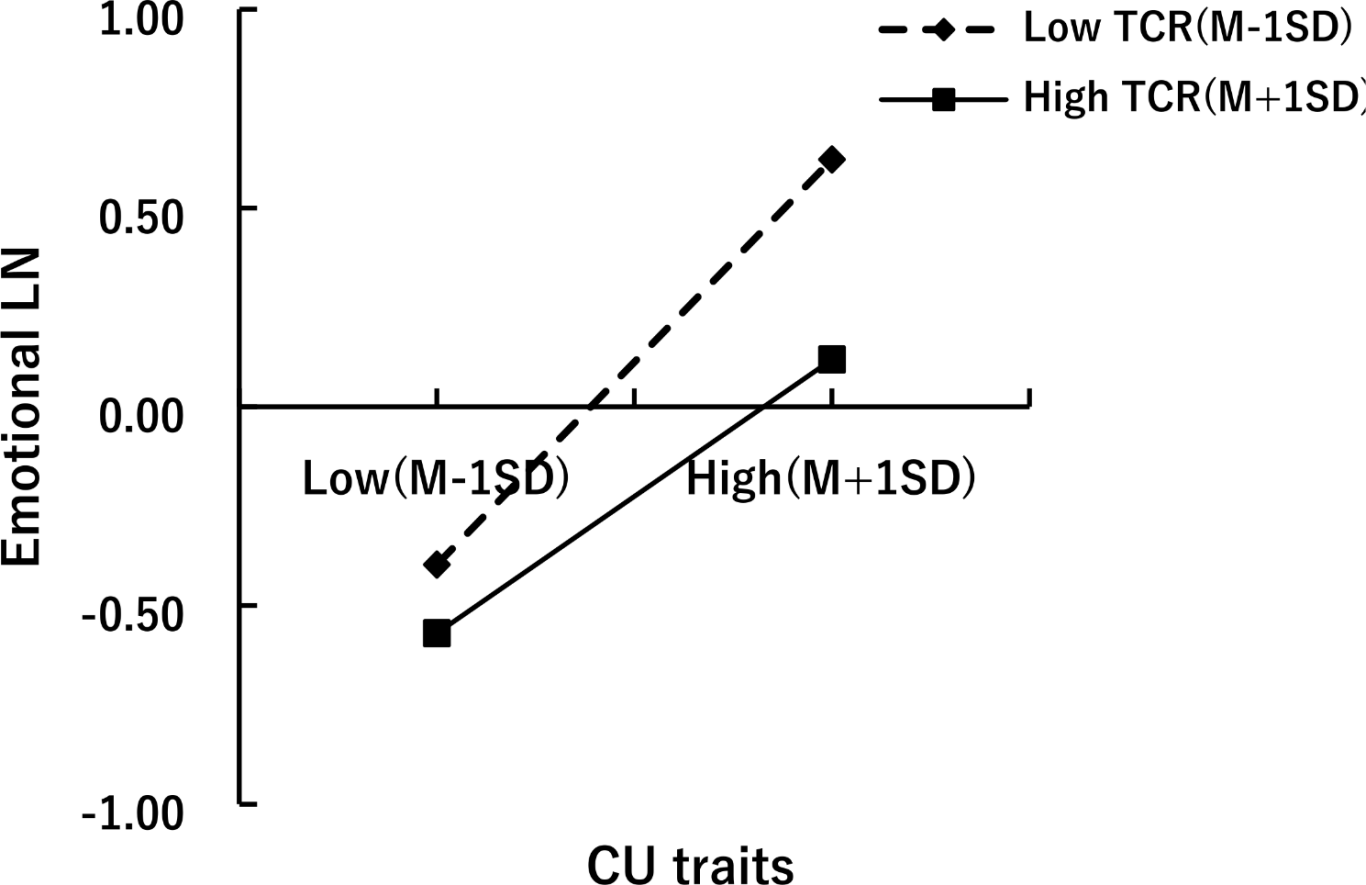
Moderating effect of positive TCR

In order to explore the moderating effect of the TCR, the score for TCR was divided into three conditions: high, medium, and low. As shown in Fig. 1, the effect values and 95% bootstrap confidence intervals for CU traits, emotional LN, and a positive TCR validated a moderated mediation model. The more positive the quality of relationship between young LBC and their teachers, the more subtle the negative effect of CU traits on emotional LN. In summary, it can be concluded that emotional LN serves as a mediator between CU traits and EPBs in preschool LBC, and the first half of the pathway is moderated by a positive TCR, which reduces the impact of CU traits on emotional LN.

## Discussion

This study explored the mechanism behind a correlation of CU traits and EPBs in preschool LBC in China. Based on existing theoretical models and empirical studies, the indirect effects of emotional LN and TCR were proposed and verified. The results showed that in left-behind children during early childhood, CU traits can significantly positively affect the occurrence of EPBs, and in this process, emotional LN plays a mediating role significantly; however, a positive TCR can reduce emotional LN in children, in turn reducing the frequency of EPBs.

Social adaptation is a topic that has been the focus of research on LBC, especially externalizing behaviors. Previous studies have explored predictors of externalizing behaviors in LBC, such as social support and personality traits [22, 79, 80]. Research involving externalizing behaviors and CU traits have been conducted mainly with school-age children and adolescents, and even adults. However, it was unclear whether CU traits would have an impact on externalizing behaviors in preschool children, especially in LBC. The results of this study revealed that CU traits profoundly influenced externalizing behaviors (Hypothesis 1), which is consistent with the results of previous studies [81–83]. This also suggests that CU traits are an important marker variable for externalizing disorders during a critical stage of personality development and early childhood socialization [53].

EPBs could have a negative effect on LBC’s academic performance and future social adjustment [84, 85]. Personality traits (e,g. CU traits) in LBC have received growing attention [8], and CU traits have been found to be predictive of social adaption and externalizing problems in early childhood [15, 53]. Consequently, the findings of this study offer new evidence of the possible effect of CU traits on LBC’s behavior during early childhood.

The association between CU traits and externalizing behaviors in preschool LBC was demonstrated to be mediated by emotional LN in this study (Hypothesis 2), which is consistent with previous research on the mediating role of emotional LN between personality traits and behavioral disorders in children [36, 86, 87]. The results support the GAM, which proposes that individual factors trigger impulsive behaviors through psychological and emotional arousal [7]. In addition, the findings provide empirical support for the STAR model [39]. Emotional LN in preschool LBC positively predicted externalizing symptoms, which is consistent with the findings of previous studies on general preschool children [42, 88]. Children with high CU traits may show impulsivity and experience negative emotionality [33], which tends to be self-oriented [11]. In other words, children with CU traits tend to ignore the feelings of others in social situations, which appears as callousness. However, when related to the self, emotions may explode due to external pressure and lack of self-control [7, 89, 90]. In addition, according to the STAR model [39], left-behind experience during early childhood contributes to an insecure attachment style with migrant parents and left-behind guardians, which may further reinforce the mediating role of emotional LN between CU traits and externalizing behaviors.

A positive TCR was negatively associated with emotional LN, consistent with previous studies [91]. Moreover, the TCR moderated the relationship between CU traits and emotional LN (Hypothesis 3). Previous studies have found TCR moderated the link between children’s temperaments and their emotional response [54, 92]. Firstly, in accordance with the GAM and existing studies [7, 12, 53], the TCR as a protective factor would buffer the negative effects of CU traits on emotional states. Secondly, based on the perspective of attachment, although LBC are disadvantaged when it comes to establishing a secure attachment to their parents, teachers can become an alternative attachment figure for the construction of positive relationships indeed [55, 60]. Thirdly, a good fit between the TCR and the child’s temperament is associated with reduced negative performance in children [48, 93]. A positive TCR acted as a moderator in the present study providing support for the goodness-of-fit model among preschool LBC.

The support of teachers could facilitate the prosocial tendencies of LBC [94]. A positive TCR would play a compensatory role among LBC in the cultural context of a supportive environment [95]. In the forming of a high-quality TCR, teachers would support emotional expression, especially in young LBC [12]. A positive TCR would involve creating a warm climate in the classroom and offering children opportunities to exercise self-control when experiencing negative emotions [96–98]. Furthermore, emotional LN was deemed as the portent of EPBs as well [42]. In this regard, changes in emotionality may be acted out and be seen. It is reasonable to enhance the level of positive TCR to reduce emotional lability in preschool children. For preschool LBC with high CU traits, a more positive TCR should be established, so that the probability of negative emotionality will decrease, and the prevalence of EPBs will be reduced. However, further research on the association of CU traits and positive TCR is needed to understand how to enhance the social and emotional development of left-behind children during early childhood.

## Conclusion

This study examined the links between CU traits and externalizing symptoms in preschool left-behind children. The results showed that CU traits could predict externalizing behaviors, and that emotional LN acted as a mediator while the TCR moderated the connection between CU traits and emotional LN. In short, the study identified CU traits as a marker of EPBs, and underscored the importance of emotional lability/negativity and establishment of a high-quality teacher-child relationship as contributing to the dynamic association between CU traits and EPBs. The teacher-child relationship deserves more attention in future interventions for preschool left-behind children.

## Data Availability

The datasets generated and/or analysed during the current study are not publicly available but are available from the corresponding author on reasonable request.

## Abbreviations

CU: Callous-unemotional
EPBs: Externalizing problem behaviors
GAM: General aggression model
LBC: Left-behind children
LN: Emotional lability/negativity
STAR: Sensitivity to threat and affiliative reward
TCR: Teacher-child relationship
